# Effects of the Rho GTPase‐activating toxin CNF1 on fibroblasts derived from Rett syndrome patients: A pilot study

**DOI:** 10.1111/jcmm.17624

**Published:** 2023-04-20

**Authors:** Camilla Cittadini, Elena Angela Pia Germinario, Zaira Maroccia, Livia Cosentino, Valeria Maselli, Lucrezia Gambardella, Massimo Giambenedetti, Marco Guidotti, Sara Travaglione, Chiara Fallerini, Alessandra Renieri, David Israel Escobar Marcillo, Laura Ricceri, Paola Fortini, Bianca De Filippis, Carla Fiorentini, Alessia Fabbri

**Affiliations:** ^1^ Biomarkers Unit, Center for Gender‐Specific Medicine Istituto Superiore di Sanità Rome Italy; ^2^ Department of Cardiovascular, Endocrine‐Metabolic Diseases and Aging Istituto Superiore di Sanità Rome Italy; ^3^ Centre for Behavioural Sciences and Mental Health Istituto Superiore di Sanità Rome Italy; ^4^ Internal Medicine Unit and Obesity Center University Hospital Policlinico Tor Vergata Rome Italy; ^5^ Department of Food Safety, Nutrition and Veterinary Public Health Istituto Superiore di Sanità Rome Italy; ^6^ Medical Genetics University of Siena Siena Italy; ^7^ Med Biotech Hub and Competence Center, Department of Medical Biotechnologies University of Siena Siena Italy; ^8^ Genetica Medica Azienda Ospedaliera Universitaria Senese Siena Italy; ^9^ Department of Environment and Health, Mechanisms Biomarkers and Models Istituto Superiore di Sanità Rome Italy; ^10^ Association for Research on Integrative Oncology Therapies (ARTOI) Rome Italy

**Keywords:** actin, CNF1, fibroblasts, mitochondria, Rett syndrome, Rho GTPases

## Abstract

The bacterial product CNF1, through its action on the Rho GTPases, is emerging as a modulator of crucial signalling pathways involved in selected neurological diseases characterized by mitochondrial dysfunctions. Mitochondrial impairment has been hypothesized to have a key role in paramount mechanisms underlying Rett syndrome (RTT), a severe neurologic rare disorder. CNF1 has been already reported to have beneficial effects in mouse models of RTT. Using human RTT fibroblasts from four patients carrying different mutations, as a reliable disease‐in‐a‐dish model, we explored the cellular and molecular mechanisms, which can underlie the CNF1‐induced amelioration of RTT deficits. We found that CNF1 treatment modulates the Rho GTPases activity of RTT fibroblasts and induces a considerable re‐organization of the actin cytoskeleton, mainly in stress fibres. Mitochondria of RTT fibroblasts show a hyperfused morphology and CNF1 decreases the mitochondrial mass leaving substantially unaltered the mitochondrial dynamic. From a functional perspective, CNF1 induces mitochondrial membrane potential depolarization and activation of AKT in RTT fibroblasts. Given that mitochondrial quality control is altered in RTT, our results are suggestive of a reactivation of the damaged mitochondria removal via mitophagy restoration. These effects can be at the basis of the beneficial effects of CNF1 in RTT.

## 1 INTRODUCTION

Rett syndrome (RTT; OMIM #312750) is a devastating neurologic disorder with unique features, dynamic clinical evolution and a wide range of manifestations. RTT is classified as a rare X‐linked dominant disorder, which mostly affects females (1 in 10,000 female births). Males are more severely affected by RTT, but they rarely survive infancy.^1^ Currently, there is no effective therapy for RTT. In its typical form, RTT patients show a normal psychomotor development up to 6–18 months of age followed by a neurological regression characterized by the loss of acquired motor, communicative and cognitive skills leading to the development of autistic‐like features, such as anxiety, emotional withdrawal, stereotypic hand movements and diminished eye contact.^2^ In addition, multisystem comorbidities occur during patients' lifespans. Gastrointestinal and orthopaedic comorbidities are reported in over 80% of individuals^3^, ^4^ and other multisystem complications render RTT a medically complex disorder, with a severe impact on patients and their families. Classic RTT is caused in about 90–95% of cases by de novo mutations in the X‐linked gene *methyl‐CpG‐binding protein 2* (*MECP2*).^2^ Mutation status is a predictor of the symptom severity, but affected females show a wide spectrum of phenotypic variations that indicate not only a genotype–phenotype correlation but also a cellular mosaicism, resulting in a multitude of cells expressing either the wild‐type or the mutant version of MeCP2.^5^ MeCP2 is ubiquitously expressed and is primarily abundant within the brain.^6^ Its mutations can impair the functionality of many genes, in both nervous and other tissues.^7^, ^8^ Although the molecular mechanisms, leading from *MECP2* gene mutations to the RTT development and progression, are not yet completely clarified,^9^ several lines of evidence strongly suggest that mitochondrial dysfunction has a causal role in RTT pathogenesis onset.^10^, ^11^, ^12^ It is already known that MeCP2 is involved in the transcriptional regulation of several genes encoding for mitochondrial factors, suggesting that *MECP2* mutations can compromise the mitochondrial functionality.^10^, ^13^ In fact, altered mitochondrial structure and deficiencies in mitochondrial enzyme activities in different cells or tissues derived from RTT patients have been described, such as elevated markers for oxidative stress, mitochondrial depolarization or a decreased ATP level (see Ref. [10] for a review). Interestingly, recent reports suggest that the mitochondrial quality control (MQC) system, which is constituted by mitochondrial dynamics, mitophagy and biogenesis, is impaired in RTT.^13^, ^14^, ^15^, ^16^

Studies on RTT have been mostly performed on MeCP2 gene knockout and defective murine models that closely mimic the clinical features of the human disorder, including mitochondrial impairment in peripheral organs described in RTT patients.^17^ Cytotoxic necrotizing factor 1 (CNF1), a protein toxin produced by some pathogenic *Escherichia coli*, has been reported to improve the behavioural phenotype and to revert the atrophy in astrocytes in a mouse model of RTT carrying a truncating mutation of the *Mecp2* gene, Mecp2‐308 mice.^18^ This RTT model, extensively used also for studies with CNF1,^12^, ^19^ shows both a later onset of symptoms and a longer life expectation than the null mutants.^20^, ^21^ CNF1 exerts its activity by modulating the Rho GTPases Rho, Rac and Cdc42,^22^, ^23^ regulatory proteins involved in the activation of a number of effectors and signalling pathways crucial for cellular life.^24^ In Mecp2‐308 mice, the modulation of Rho GTPases reverses the alterations of Rho protein‐dependent signalling pathways critically involved in the regulation of actin‐dependent cytoskeletal remodelling and protein translation, key biological processes impaired in RTT mouse brain. In particular, CNF1 reshapes the actin cytoskeleton and enhances neurotransmission and synaptic plasticity in mouse brains.^18^ Interestingly, CNF1 also counteracts the RTT‐related mitochondrial defects restoring the mitochondrial respiratory chain complexes and ATP synthase activities, known to be crucial players for the cell energy production.^12^, ^19^, ^25^ As a whole, these preclinical data suggest a potential role for Rho GTPases as therapeutic targets for RTT.

Given the results obtained in RTT mouse models by CNF1 treatment, we herein analyse the effect of CNF1 on fibroblasts from four RTT patients carrying four different mutations exemplifying the wide spectrum of RTT mutations. This genetically heterogeneous cellular system summarizes various aspects of RTT pathophysiology, thus representing a valuable model for cellular and molecular investigations that would permit a deeper understanding of the genetic landscape of the RTT spectrum.^26^ In particular, the morphological effects and mitochondrial responses, as well as the involvement of AKT, already proven to be activated by the toxin,^27^ and the autophagic markers were analysed to further characterize the beneficial role of CNF1 in a pilot study on fibroblasts isolated from RTT patients.

## 2 MATERIALS AND METHODS

### 2.1 Cells, cultures, treatments and inhibitors

Fibroblasts, provided by the ‘Cell lines and DNA bank of Rett syndrome, X‐linked mental retardation and other genetic diseases’, which is part of the Genetic and COVID‐19 Biobank of Siena, Italy, member of BBMRI‐IT, of Telethon Network of Genetic Biobanks (project no. GTB18001), of EuroBioBank and of D‐Connect, were from four RTT patients carrying four different mutations: (1) RTT1, carrying *MECP2* late truncating mutation (*MECP2* A443X); (2) RTT2, carrying a deletion of exons 3 and 4; (3) RTT3, with a preserved speech variant; (4) RTT4, carrying *MECP2* late truncating mutation (*MECP2* R294X) (data summarized in Figure S1). Human primary fibroblasts from healthy donors were used as WT control fibroblasts. Cells were cultured in high glucose Dulbecco's Modified Eagle's medium (Gibco, Carlsbad), supplemented with 20% foetal bovine serum (FBS GE Healthcare), penicillin (100 U/ml) and streptomycin (100 μg/mL) (Gibco).

CNF1 was purified from the hyperproductive *E. coli* strain pISS392 (kindly provided by V. Falbo, Istituto Superiore di Sanità). The mutant CNF1 (mCNF1), devoid of enzymatic activity due to the replacement of cysteine to serine at position 866,^28^ was purified from the plasmid coding for CNF1 C866S (kindly provided by E. Lemichez, Inserm). For most experiments, cells were seeded at a density of 2 × 10^5^ cells/cm^2^ and, 24 h after the seeding, cells were treated with 10^−10^ M CNF1 or mCNF1 for 4, 24 and 48 h.

### 2.2 Fluorescence microscopy

Control (untreated or mCNF1‐treated) and CNF1‐treated fibroblasts, seeded on glass coverslips, were fixed with 3.7% paraformaldehyde (Carlo Erba), permeabilized with Triton 0.5% X‐100 (Sigma‐Aldrich, St. Louis) and then incubated at 37°C for 30 min with Tetramethylrhodamine‐isothiocyanate (TRITC)‐phalloidin (Sigma‐Aldrich, cat. n. P1951), diluted 1:100 in PBS for actin cytoskeleton staining. Nuclei were stained for 5 min at RT with 0.2 μg/ml Hoechst 33258 (Sigma‐Aldrich, cat. n. 94,403). To stain mitochondria, cells were incubated with 1 μM Mitotracker Red CMXRos (Invitrogen, Waltham, cat. n. M7512), 30 min before fixation with paraformaldehyde. To stain p62, cells were incubated with Anti‐p62/SQSTM1primary antibody (Sigma‐Aldrich, cat. n. P0067) for 30 min and, following extensive washing, incubated with the appropriate secondary antibody (Alexa Fluor Thermofisher Scientific Invitrogen, cat n. A‐11034). Finally, glass slides were observed with an Olympus BX51/BX52 fluorescence optical microscope (Olympus Corporation of the Americas, Center Valley) equipped with a charge‐coupled device camera (Carl Zeiss). Images were acquired using the IAS 2000 (Delta Systems Inc., Streetsboro) programme.

### 2.3 Protein extraction and western blot analysis

Cells were lysed (50 mM Tris–HCl pH 6.8, 2% SDS, 10% glycerol), and protein concentration was determined by using the ‘Bio‐Rad Protein Dc assay’ (Bio‐Rad Laboratories, Hercules). Twenty μg of total proteins were separated on SDS‐PAGE polyacrylamide gel and subsequently electrically transferred onto polyvinylidene difluoride membranes (Bio‐Rad Laboratories) filters. After saturating the free sites with TBS‐T (20 mM Tris–HCl pH 7.5, 150 mM NaCl, 0.02% Tween 20) containing 5% skimmed milk (Bio‐Rad Laboratories), membranes were incubated overnight at 4°C with the following antibodies diluted in TBS‐T containing 5% skim milk or 5% BSA (Sigma‐Aldrich): anti‐OPA1 mouse monoclonal antibody (1:500, BD Biosciences, San Jose, cat. n. 612,006); anti‐RhoA mouse monoclonal antibody (1:500, Santa Cruz Biotechnology, Santa Cruz, cat. n. sc‐418); anti‐Rac1 mouse monoclonal antibody (1:500, BD Biosciences, cat. n. 610,650); anti‐Mfn2 rabbit polyclonal antibody (1:1000, Cell Signaling Technology, Boston, cat. n. 94,825); anti‐GAPDH mouse monoclonal antibody (1:500, Santa Cruz, cat. n. sc‐32,233); anti‐Drp1 mouse monoclonal antibody (1:1000, BD Biosciences, cat. n. 611,113); anti‐phosphoDrp1 (pDrp1) rabbit polyclonal antibody (1:1000, Cell Signaling Technology, cat. n. 4867 s); anti‐AKT Pan rabbit polyclonal antibody (1:1000, Invitrogen, cat. n. 44‐609G), anti‐phospho‐AKT ser 473 rabbit monoclonal antibody (1:1000, Invitrogen, cat. n. 44‐623G), anti‐p62/SQSTM1 rabbit polyclonal antibody (1:1000, Sigma‐Aldrich, cat. n. P0067), anti‐LC3 a/b rabbit polyclonal antibody (1:1000, Cell Signaling Technology, cat n. 4108) and anti‐PINK1 rabbit monoclonal antibody (D8G3) (1:500, Cell Signaling Technology, cat. n. 6946).

After washing in TBS‐T, immunocomplexes were detected with Horseradish peroxidase‐conjugated species‐specific secondary antibodies (Jackson Laboratories, Bar Harbor), followed by enhanced chemiluminescence reaction (Millipore Corporation, Billerica). Reactive bands were detected by the ChemiDoc MP system (Bio‐Rad Laboratories), and signal quantification was performed using the IMAGE LAB software (Bio‐Rad Laboratories). Proteins were normalized as a function of α‐tubulin or GAPDH. pDrp1 was normalized as a function of total Drp1.

### 2.4 Activated Rho GTPases pull‐down assay

Pull‐down assay was performed as previously described.^27^ Briefly, cells were lysed in the appropriate buffer and cleared lysates were incubated with 80 μg of glutathione S‐transferase (GST) Rhotekin (for Rho; Cytoskeleton, Denver, cat. n. BK‐036) and GST‐PAK‐CD (for Rac) fusion proteins, bound to glutathione‐coupled Sepharose beads (GE Healthcare, Little Chalfont, cat. n. 17,098,903) for 40 min at 4°C, in rotation.

Following extensive washing, bound proteins were resuspended in sample buffer and subjected to SDS‐PAGE as described above. Two percentage of the cell lysate used in the assay was analysed in parallel, and the amount of the activated Rho GTPases was normalized as a function of the amount of total protein used for each sample.

### 2.5 Flow cytometry

Cytometric analyses were used to measure mitochondrial membrane potential and mitochondrial status. For both measurements, 5 × 10^5^ cells were seeded and maintained under the conditions described above. At each time point, following washing in 1X PBS, the mitochondrial membrane potential was analysed in controls and CNF1‐treated fibroblasts by using 10 μM of JC‐1 (5‐5′,6‐6′‐tetrachloro‐1,1′,3,3′‐tetraethyl benzimidazole‐carbocyanine iodide probe; Invitrogen, cat. n. T3168) for 15 min at 37°C in 1X PBS. For mitochondrial status, 1 μM Mitotracker Green FM (Molecular Probes, cat. n. M7514) was used for 30 min at 37°C in 1X PBS. Then, cells were analysed on a FACScalibur cytometer (BD Biosciences) equipped with a 488 argon and with a 635 red diode laser. Data were recorded and statistically analysed by a Macintosh computer using CellQuest software (BD Biosciences). The expression level of the analysed probes was expressed with median fluorescence for Mitotracker Green FM and with percentage of cells with depolarized or hyperpolarized mitochondria for JC‐1.

### 2.6 DNA Isolation, Droplet digital PCR and Real‐time PCR

Total DNA was isolated from human fibroblasts. In detail, 5 × 10^5^ cells were seeded and subsequently treated with CNF1. The cells were harvested at different times, and the pellets were collected by centrifugation (300 g, 5 min, 4°C). QIAamp DNA (Qiagen, Hilden, cat. n. 51,326) kit was used to isolate total DNA accordingly to the manufacturer's instructions. Relative mitochondrial DNA copy number was analysed by both absolute QPCR (Life Technologies 7500 Fast Real Time System; Austin) and ddPCR (Bio‐Rad QX 200). Mitochondrial and nuclear DNA was detected by using ND2 and Rplp0 single tube Taqman real‐time PCR assay, respectively (Life Technologies, Austin, cat. n. 4,331,182).

### 2.7 Statistical analysis

Statistical analyses were performed using IBM SPSS Statistics for Windows (IBM Corp. Version 25.0. Armonk), while graphs were obtained using ggplot2 package in R version 3.6.2. All values shown in the graphs correspond to the mean ± SEM or median (interquartile range) ±1.5*interquartile range of three separate experiments performed in duplicate. Normality and homoscedasticity were evaluated using Shapiro–Wilk's and Breusch–Pagan's tests, respectively. One‐way anova or Kruskal–Wallis test was used for fibroblast data. Post hoc comparisons were performed using Dunnett's or Dunn's tests (setting the untreated group as control). Animals were excluded from the analyses when identified as outliers by the use of the Grubbs' test or the interquartile range method. A *p* Value <0.5 was considered significant.

## 3 RESULTS

### 3.1 CNF1 induces RhoGTPases activation and actin cytoskeleton modification in RTT fibroblasts

**FIGURE 1:**
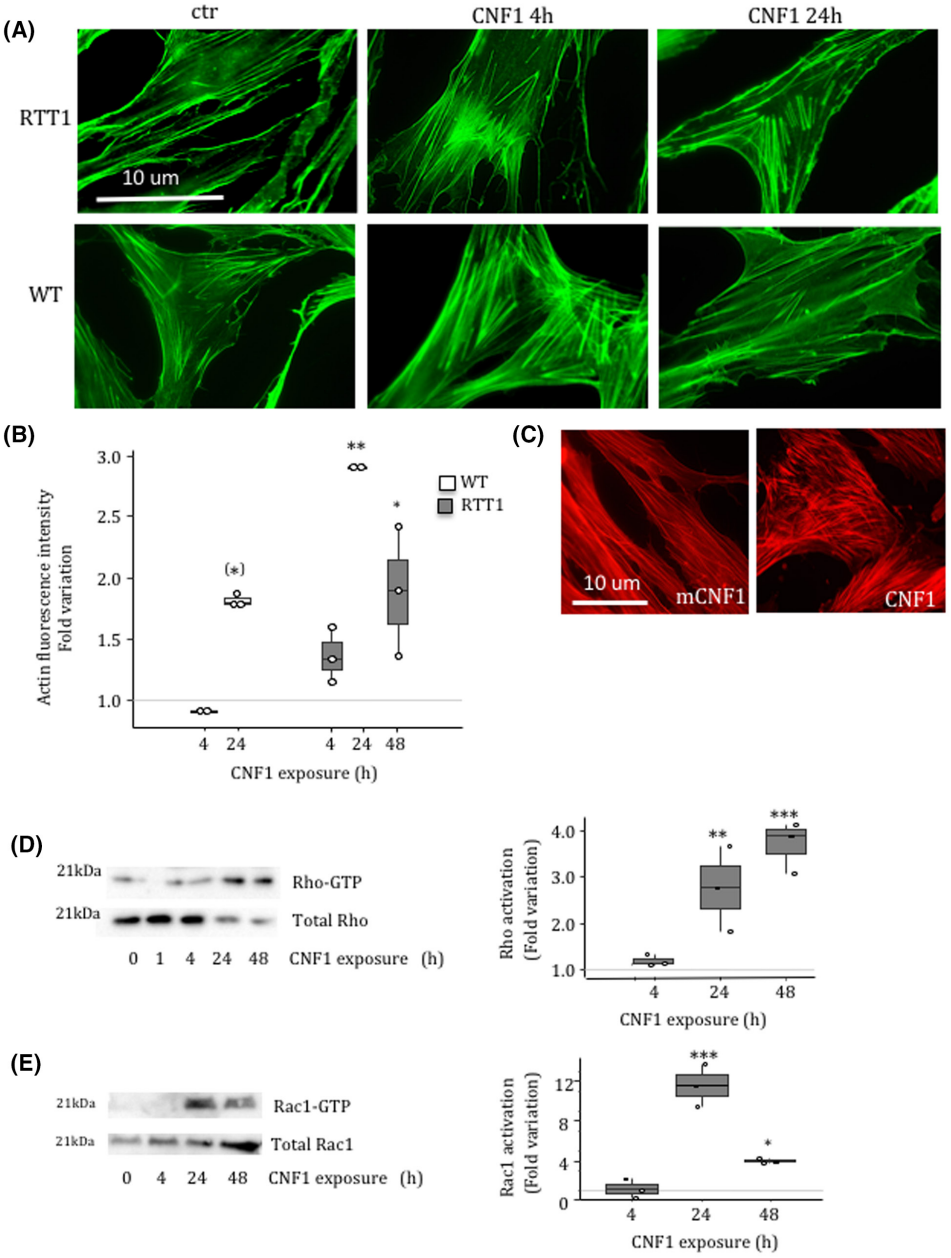
Effects of CNF1 on RTT fibroblasts cytoskeleton. (A) Fluorescence micrographs of WT and RTT1 fibroblasts treated with CNF1 for 4 and 24 h and stained with FITC‐phalloidin to visualize the actin cytoskeleton. (B) Graph showing the actin fluorescence intensity (Fold activation). (C) Fluorescence micrographs of CNF1‐ and mCNF1‐treated RTT1 cells stained with TRITC‐phalloidin. (D and E) Immunoblot showing the amount of activated Rho (D) and Rac (E) after different times of CNF1 exposure, obtained by pull‐down assays of fibroblasts from patient RTT1. Immunoblots were analysed by densitometry, as described in Materials and Methods. The graphs (right panels) report the relative activity of Rho/Rac normalized as a function of the total amount of Rho/Rac protein loaded. Data are expressed relative to the values of untreated cells (control = 1, dashed line). Data are median (interquartile range) ±1.5*interquartile range. Statistical significance was assessed using One‐way anova + Dunnett's post hoc test or Kruskal–Wallis + Dunn's post hoc tests. (*) *p* < 0.1; *p < 0.05; **p < 0.01; ***p < 0.001.

Primary cultured fibroblasts derived from RTT patients were treated with CNF1 10^−10^ M for 4 and 24 h and then underwent fluorescence microscopy analysis after staining with FITC‐phalloidin. As shown in Figure 1A (upper panels and in Figure S2A), whereas untreated RTT fibroblasts contained a few thin stress fibres, treatment with CNF1 for 4 and 24 h induced a considerable re‐organization of F‐actin in prominent and thick stress fibres. The same modification of the actin cytoskeleton was observed in WT fibroblasts (Figure 1A, lower panels). Both WT and RTT fibroblasts showed an increase in fluorescence intensity starting at 24 h of toxin exposure (Figure 1B and Figure S2B).

To verify the causal role of CNF1 on RTT fibroblasts, we exposed cells to the catalytic inactive variant of CNF1, mCNF1 that is devoid of action on the Rho GTPases. As shown in Figure 1C, the actin cytoskeleton organization of fibroblasts exposed for 48 h to mCNF1 strongly resembles that of control cells, whereas a marked increase in stress fibres was evident in cells exposed to CNF1. Being mCNF1 unable to modify the cytoskeletal architecture in RTT fibroblasts, these results clearly evidence that the capacity of the toxin to remodel the cytoskeleton in RTT fibroblasts is strongly linked to its enzymatic activity on the Rho GTPases. We then quantified Rho and Rac1 activities in the presence of CNF1 by pull‐down experiments. Figure 1D,E shows that the relative activity of both GTPases increased in RTT fibroblasts starting from 24 h of treatment. However, while the Rho protein remained strongly activated until 48 h (Figure 1D), the level of Rac1‐GTP decreased at 48 h, although the protein persisted in an active state (Figure 1E).

### 3.2 CNF1 decreases the mitochondrial mass in RTT fibroblasts

**FIGURE 2:**
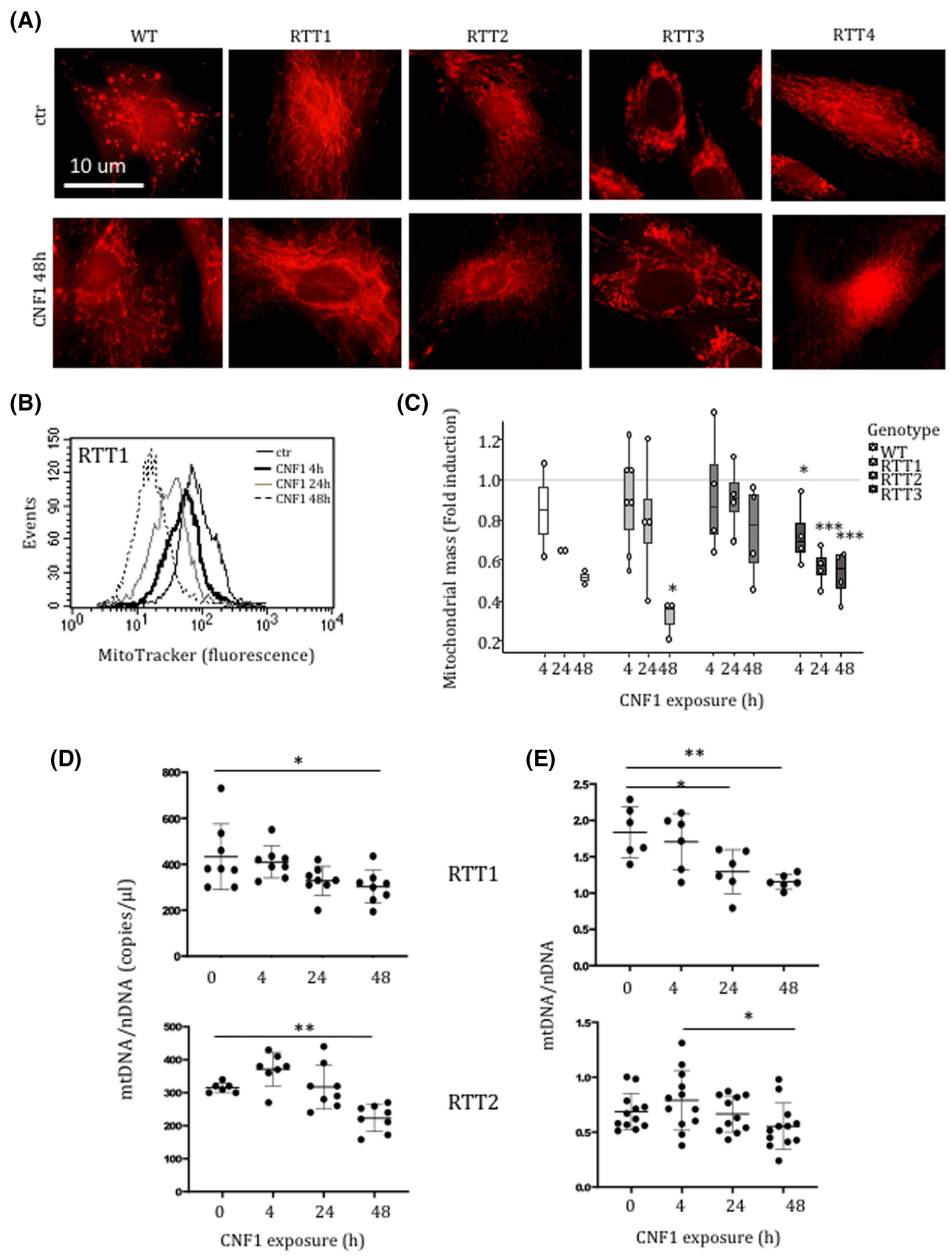
CNF1 reduces mitochondrial mass in RTT fibroblasts. (A) Fluorescence micrographs of WT and RTT fibroblasts treated with CNF1 and stained with Mitotracker Red to visualize mitochondria. (B and C) Flow cytometry analysis of Mitotracker Green: (B) representative image and (C) graph showing the intensity of fluorescence. (D) Digital droplet PCR and (E) real‐time PCR showing the ratio of the mtDNA/nDNA in RTT1 and RTT2 fibroblasts treated with CNF1. Data in (C) are expressed relative to the values of untreated cells (control = 1, dashed line). Data are mean ± SEM or median (interquartile range) ±1.5*interquartile range. Statistical significance was assessed using One‐way anova + Dunnett's post hoc test or Kruskal–Wallis + Dunn's post hoc tests. *p < 0.05; **p < 0.01; ***p < 0.001.

In previous studies, we have shown that CNF1 impacts mitochondrial morphology and function in vitro, although in a cell type‐dependent manner,^29^, ^30^, ^31^ and in a mouse model of RTT.^12^, ^19^ Thus, we analysed the effects of CNF1 on mitochondria in RTT patients' fibroblasts. The mitochondrial dye Mitotracker was used to visualize mitochondria by fluorescence microscopy. RTT fibroblasts showed a varied morphology, with cells of some patients displaying a filamentous interconnected network of mitochondria, as already reported,^14^ and others showing a dot‐like morphology (Figure 2A). Exposure to CNF1 only slightly modified the phenotype, with mitochondria appearing only moderately elongated in treated fibroblasts. Surprisingly, when a quantitative analysis of cells stained with Mitotracker Green was performed by flow cytometry, we found that the fluorescence intensity decreased as a function of CNF1 exposure duration (Figure 2B,C). Consistently, the mitochondrial DNA and nuclear DNA (mtDNA/nDNA) ratio was reduced (Figure 2D,E), strongly suggesting a reduction of the mitochondrial mass.

Altogether, these results indicate that CNF1 induces a loss in mitochondrial mass in RTT fibroblasts, probably due to the removal of dysfunctional mitochondria.

### 3.3 CNF1 modifies the mitochondrial functionality and induces autophagic markers in RTT fibroblasts

**FIGURE 3:**
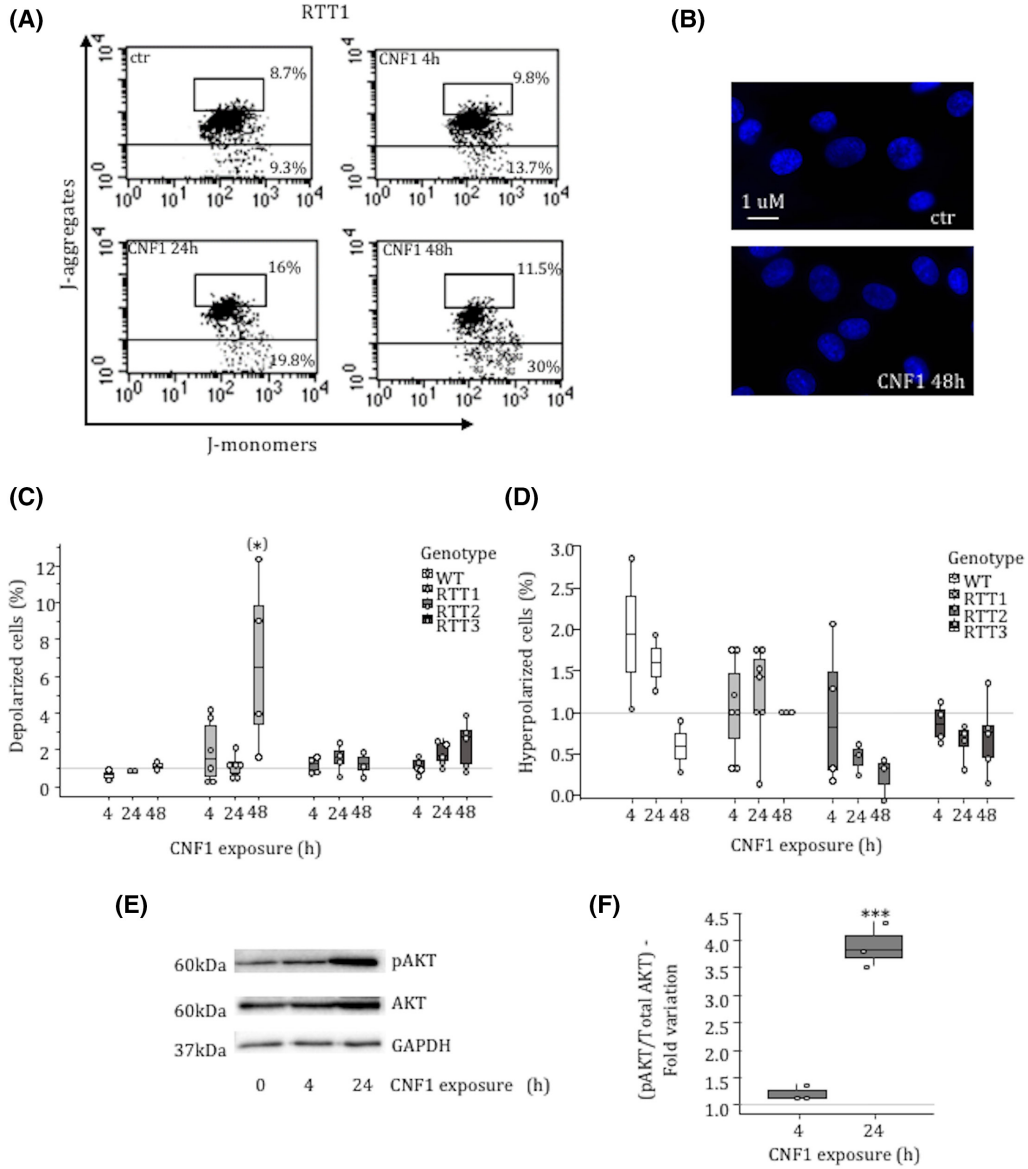
CNF1 activity on mitochondrial functionality. (A) Flow Cytometry analysis of JC‐1 stained cells. (B) Fluorescence micrographs of RTT1 fibroblasts stained with Hoechst 33258 to visualize nuclei. (C and D) Graph showing the percentage of (C) depolarized and (D) hyperpolarized WT and RTT fibroblasts following CNF1 exposure, as obtained by flow cytometry analysis. (E and F) Western blot analysis of phosphor‐AKT (pAKT) in whole‐cell lysates of RTT1 fibroblasts after treatment with CNF1. Data are expressed relative to the values of untreated cells (control = 1, dashed line). Data are median (interquartile range) ±1.5*interquartile range. Statistical significance was assessed using One‐way anova + Dunnett's post hoc test or Kruskal–Wallis + Dunn's post hoc tests. Data are mean ± SEM. Statistical significance was assessed using the Kruskal–Wallis test. (*) *p* < 0.1; ***p < 0.001.

According to the different cellular stimuli and metabolic demands, mitochondria are regulated by four high molecular weight GTPases, namely the mitofusins (Mfn1 / Mfn2) and OPA1 that control their fusion, Drp1 and hFis1 that regulate their fission. In contrast with our previous data obtained in other cell systems, showing that CNF1 can increase the amount of the regulatory proteins involved in mitochondrial dynamics,^16^, ^29^, ^32^ no changes could be observed in fibroblasts from RTT1 patients (Figure S2). We thus investigated the functionality of mitochondria focusing on mitochondrial membrane potential (Figure 3A). In RTT fibroblasts treated with CNF1, a slight portion of cells underwent mitochondrial membrane depolarization after 48 h of toxin exposure (Figure 3C) whereas no hyperpolarization was evidenced (Figure 3D). No apoptotic fibroblasts were evidenced by nuclear staining with Hoechst (Figure 3B) and cells remained viable until at least 10 days (data not shown). On the contrary, in WT fibroblasts CNF1 was able to elongate mitochondria (Figure 2A), to significantly increase both OPA1 and Mfn2 (Figure S2) and no signs of either depolarization of the mitochondrial membrane potential were found (Figure 3C,D).

**FIGURE 4:**
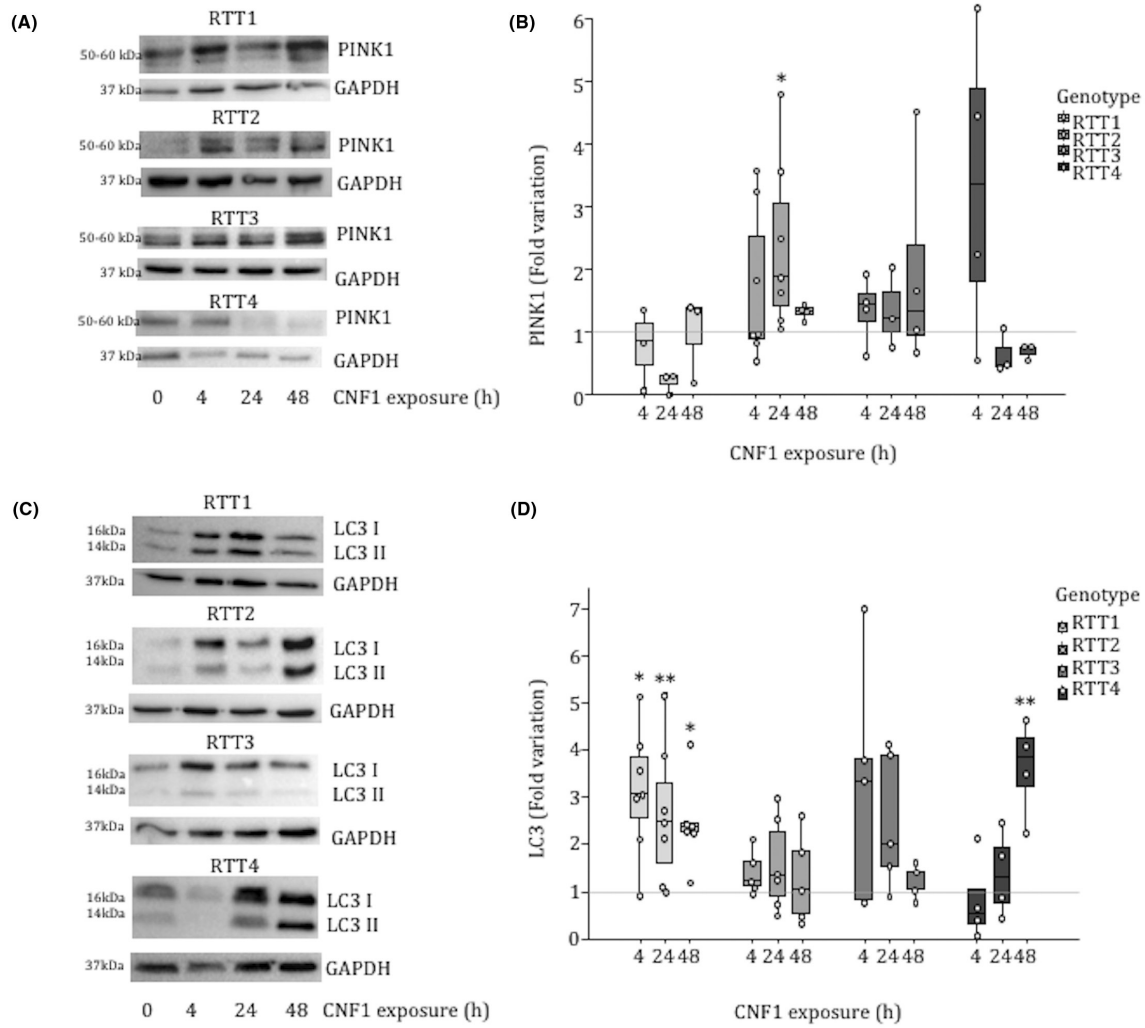
CNF1 induces autophagic markers in RTT fibroblasts. Western blot analysis of the autophagic markers (A) PINK1 and (C) LC3. In the boxplots (B and D), PINK1 and LC3 were normalized as a function of the GAPDH protein. Data are expressed relative to the values of untreated cells (control = 1, dashed line). Data are median (interquartile range) ±1.5*interquartile range. Statistical significance was assessed using One‐way anova + Dunnett's post hoc test or Kruskal–Wallis + Dunn's post hoc tests. Data are mean ± SEM. Statistical significance was assessed using the Kruskal–Wallis test. *p < 0.05; **p < 0.01.

**FIGURE 5:**
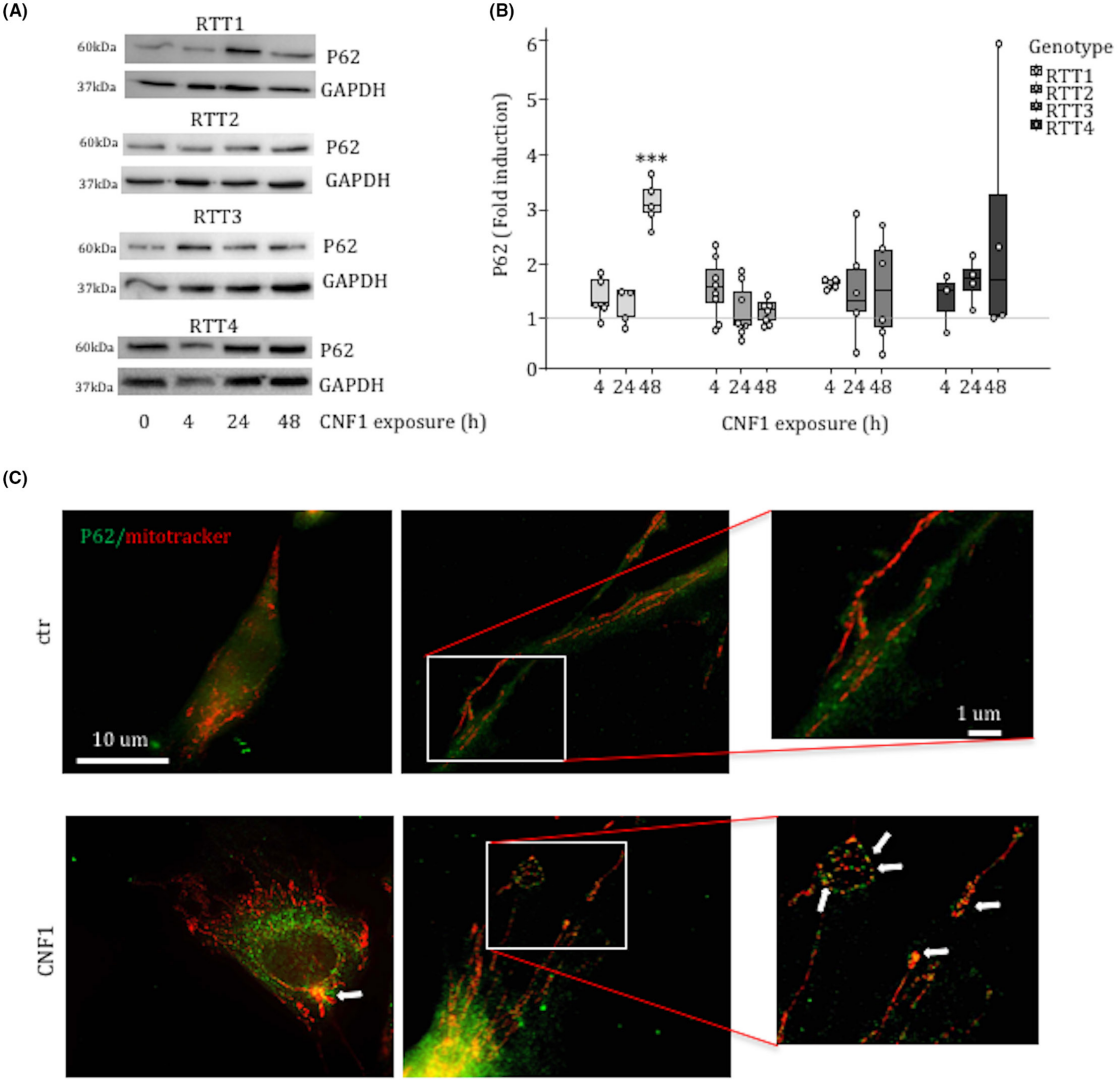
p62 co‐localizes with small mitochondria in CNF1‐treated RTT fibroblasts. Western blot analysis of the autophagic markers p62 (A) with the respective boxplots (B) normalized as a function of the GAPDH protein. Fluorescence micrographs of control and CNF1 treated RTT1 fibroblasts showing colocalization of the autophagic marker p62 and mitochondria (C). Insets show a magnification of small mitochondria colocalized with p62. Data are expressed relative to the values of untreated cells (control = 1, dashed line). Data are median (interquartile range) ±1.5*interquartile range. Statistical significance was assessed using One‐way anova + Dunnett's post hoc test or Kruskal–Wallis + Dunn's post hoc tests. ***p < 0.001.

Our results, indicating a reduction of the mitochondria mass, point out to a selective activation of the mitochondrial quality control (MQC) system in RTT fibroblasts after CNF1 exposure. It is well established that AKT activation stimulates mitophagy via the recruitment of the E3 ubiquitin ligase Parkin and PINK1 (PTEN‐induced kinase 1) to depolarized mitochondria.^33^, ^34^ We have previously reported that, in epithelial cells, the CNF1‐induced activation of the Rho GTPases stimulates the PI3K/Akt pathway eventually modifying the architecture of the mitochondrial network.^27^ Consistently, the activation of PI3K/Akt pathway was also observed in RTT1 fibroblasts after exposure to CNF1, which leads to a significant increase of pAKT 24 h after toxin exposure (Figure 3E,F). Then, we evaluated whether CNF1 was able to modulate the autophagic markers. RTT fibroblasts exposed to CNF1 showed an increased amount of PINK1 (Figure 4A,B) as well as LC3 (Figure 4C,D), the crucial biological marker used to identify autophagy in mammalian systems. Both forms of LC3 were increased by CNF1 in all the RTT fibroblasts analysed although at different time points. In addition, the autophagy receptor sequestosome 1 (SQSTM1/p62), another widely used marker for autophagy, was increased in RTT fibroblasts exposed to CNF1 (Figure 5A,B), indicating a defective autophagic flux. To substantiate the involvement of p62 in the CNF1‐induced effects on mitochondria from RTT patients, we performed a double immunostaining of p62 and Mitotracker (Figure 5C and Figure S3) and we found that p62 actually co‐stained with Mitotracker in mitochondria and was primarily identified at either perinuclear region or cell peripheral region.

## 4 DISCUSSION

Despite the extensive studies on RTT during the last years, the molecular and pathogenic mechanisms by which *MECP2* mutation drives pathology in RTT patients remain unclarified. Several metabolic components contribute to the phenotypical manifestations of RTT,^16^, ^35^ thus making difficult the analysis of RTT development and progression. Rho GTPases modulation by the bacterial product CNF1 has been reported to ameliorate the cognitive deficits and to rescue the mitochondrial defects *in* in vivo models of RTT,^12^, ^18^, ^25^ thus suggesting CNF1 as a promising therapeutic drug. However, the molecular and cellular mechanisms which underlie the beneficial effects observed in the RTT models after exposure to CNF1 were still unclear. In this pilot study, we have used in vitro cultures of primary fibroblasts isolated from four RTT patients carrying different mutations as a human reliable disease‐in‐a‐dish model.^26^ Our results indicate that, in line with our previous results in an RTT mouse model, CNF1 can modulate Rho GTPases and hence polymerize the actin cytoskeleton in human fibroblasts too. In addition, CNF1 decreases the mitochondrial mass, stimulates mitochondrial depolarization, activates AKT phosphorylation and increases autophagic markers suggesting a global enhancement of the MQC.

CNF1 is able to polymerize the actin cytoskeleton in RTT primary cultured fibroblasts herein tested and to modulate the small GTPases Rho and Rac, the actual target of CNF1.^22^, ^23^ CNF1 is known to cause actin cytoskeleton polymerization in all the model systems tested so far, including neuronal cells.^36^, ^37^ Cytoskeleton plays a pivotal role in controlling brain structural plasticity, axons, dendrites and spines formation and cytoskeletal alterations may be at the basis of cognitive impairments.^38^ The actin cytoskeleton is controlled by the small GTPases of the Rho family, and Rho proteins are considered essential factors in linking synaptic plasticity, neuronal spines formation and cognitive abilities.^38^ Since pharmacological studies depict an unclear role for RhoA in cognitive function,^39^ we cannot exclude that the persistent activation of RhoA observed in our study may also play a role in a beneficial effect of CNF1 in in vivo models.^12^, ^18^ On the contrary, the CNF1‐induced activation/deactivation rate of Rac1 observed in fibroblasts may physiologically stimulate crucial cellular pathways. Indeed, Rac1 is associated and necessary for the development of cognitive capabilities, an optimal level of Rac1 likely leading the way to normal neuronal morphology during activity‐dependent plasticity.^40^, ^41^, ^42^, ^43^, ^44^ In agreement, Rac1 has been found upregulated in several neurodevelopmental disorders^42^ including RTT. Thus, Rac1 activation and function are becoming an important therapeutic target to rescue disorders associated with cognitive impairment.^39^, ^45^ The ability of CNF1 to modulate the Rac1 protein in fibroblasts from RTT patients let us speculate on a role for CNF1 to rescue or mitigate behavioural and neurological deficits. Indeed, although the results herein reported are limited to fibroblasts, our results suggest that the cytoskeletal modification and the Rho GTPases activation may also play a role in the improvement of the behavioural phenotype induced by CNF1 in the RTT model.^18^

It is interesting to note that the modulation of Rho GTPases by CNF1 has been described to rescue mitochondrial dysfunctions in Mecp2‐308 mice and null mice.^12^, ^25^ Various lines of evidence point to mitochondria dysfunction as a possible contributing factor in RTT pathogenesis. In the last years, several reports documented an altered mitochondrial structure, as well as deficiencies in mitochondrial enzyme activity in different RTT‐derived cells or tissues.^10^, ^13^, ^46^ Such alterations could be profoundly improved by mitochondrial acting drugs.^16^, ^47^ Crivellari and co‐workers^14^ reported that RTT fibroblasts display elongated and interconnected mitochondria, and we herein found that the addition of CNF1 only slightly enhanced their architecture in all the patients' fibroblasts tested. It is well known that in different cell lines CNF1 causes mitochondria elongation,^27^ due to fission inhibition and to a shift of the balance of mitochondrial dynamics towards unopposed fusion.^29^ This phenomenon results also in the promotion of mitochondrial activity in terms of increased cellular ATP content, probably due to an increment of the mitochondrial electron transport chain in several systems.^29^ Unexpectedly, we have found that in RTT fibroblasts, CNF1 causes a time‐dependent decrease in Mitotracker staining and in the ratio of mtDNA/nDNA, indicating a reduction in mitochondrial mass. One possibility is that CNF1 may stimulate the decrease in mitochondrial mass by promoting the elimination of damaged mitochondria. It is interesting to note that recent reports suggest that the MQC system is compromised in RTT. Crivellari and co‐workers showed, in fact, that in RTT fibroblasts mitophagy is impaired, mitochondria are hyperfused with increased volume and mitochondrial fusion and mitophagy genes expression is altered.^13^, ^14^, ^15^ In control fibroblasts, CNF1 significantly increases the amount of the fusion proteins OPA1 and Mfn2, as already reported for other cell types.^29^, ^32^ However, in RTT fibroblasts, CNF1 does not modify the amount of OPA1 and Mfn2 proteins and of p‐DRP1. CNF1‐dependent increase in OPA1 and Mfn2 proteins is not a general response to the toxin^31^ and the reported effects on RTT fibroblasts can be part of the CNF1‐dependent stimulation of MQC. In fact, having small mitochondria is a prerequisite for the mitophagy of damaged organelles to occur. RTT is characterized by a combination of dysregulated immune system with a persistent abnormal redox imbalance, the so‐called oxInflammation, which can account for a mitochondrial persistent damage.^48^ Mitochondrial damage leads to the loss of membrane potential (Δψm) that allows the accumulation of PINK1 on the outer mitochondrial membrane where it recruits the ubiquitin ligase, Parkin. Interaction of PINK and Parkin serves as a signal for mitophagy leading to degradation of damaged mitochondria.^49^ p62 interacts with both ubiquitin and the autophagic machinery LC3^50^, ^51^, ^52^, ^53^ linking Parkin‐catalysed ubiquitylation with mitochondrial degradation via autophagy. AKT signalling is involved in many cellular processes, including PINK1 and Parkin accumulation in response to mitochondria depolarization.^54^ Interestingly, CNF1 induces AKT phosphorylation, a decrease in Δψm and upregulation of the autophagic markers PINK, LC3 and p62 in RTT fibroblasts. p62 is frequently reported to be downregulated in the autophagic flux, and surprisingly our results demonstrate its increase. However, recent reports indicate that p62 is not essential for mitophagy despite having a significant impact on early mitophagy including PINK1 and parkin recruitment, and phosphorylation of ubiquitin on depolarized mitochondria.^55^ We have to underline that all the phenomena herein described occur in fibroblasts of different patients at different rates, thus not always reaching statistical significance. The variability of the fibroblast response is due to the fact that they derive from patients with several mutations different in size and position that cover the wide spectrum of RTT mutations. In fact, although RTT disorder is caused by mutations in a single gene, disease severity in affected individuals can be quite variable, probably due to the type of *MECP2* mutation.^56^, ^57^

Altogether, these results suggest that treatment of RTT fibroblasts with CNF1 can stimulate the removal of dysfunctional mitochondria by mitophagy. Most likely, the activation of the proteasome system, necessary to lower high levels of activated Rho GTPase caused by CNF1,^58^ makes the cells prone to intervene where a dysregulation of the mechanisms of MQC occurs. Thus, we can conceive that, following the activation of Rho GTPases, CNF1 activates AKT and contemporary causes mitochondrial membrane depolarization through a still unknown mechanism. In turn, activated AKT is known to recruit to depolarized mitochondria PINK1 and Parkin proteins to regulate downstream mitophagy. The upregulation of the autophagic markers PINK1, LC3 and p62 observed in RTT fibroblasts, although to a different extent, is in line with the hypothesis of a reactivation of the MQC. In this context, we cannot rule out the possibility that the actin cytoskeleton modification induced by CNF1 can participate in the segregation of damaged mitochondria.^59^ Although future studies are necessary to further support our hypothesis, we can speculate that this activity could be at the basis of the beneficial effects of CNF1 reported on RTT as well as on other diseases associated with mitochondrial dysfunction.^60^

## AUTHOR CONTRIBUTIONS

**Camilla Cittadini:** Investigation (lead); methodology (lead); writing – original draft (lead). **Elena Germinario:** Investigation (lead); methodology (lead); writing – original draft (lead). **Zaira Maroccia:** Writing – original draft (equal); writing – review and editing (equal). **Livia Cosentino:** Formal analysis (equal); methodology (equal). **Valeria Maselli:** Investigation (lead); methodology (lead). **Lucrezia Gambardella:** Investigation (lead); methodology (lead). **Massimo Giambenedetti:** Methodology (equal). **Marco Guidotti:** Methodology (equal). **Sara Travaglione:** Methodology (equal); writing – review and editing (equal). **Chiara Fallerini:** Investigation (lead); writing – review and editing (equal). **Alessandra Renieri:** Investigation (equal); writing – review and editing (equal). **David Israel Escobar Marcillo:** Investigation (lead); writing – review and editing (equal). **Laura Ricceri:** Methodology (equal); writing – review and editing (equal). **Paola Fortini:** Investigation (lead); writing – review and editing (equal). **Bianca De Filippis:** Data curation (equal); formal analysis (equal); funding acquisition (equal); project administration (equal); supervision (equal); writing – review and editing (equal). **Carla Fiorentini:** Writing – review and editing (equal). **Alessia Fabbri:** Conceptualization (lead); data curation (lead); formal analysis (lead); funding acquisition (equal); project administration (lead); supervision (lead); writing – original draft (lead); writing – review and editing (lead).

## FUNDING INFORMATION

This work was supported by grants from the Jerome Lejeune Foundation, France (to Alessia Fabbri), by the International Rett syndrome Foundation (#3107), USA (to Bianca De Filippis), by the Italian Ministry of Health (#GR‐2018‐12,366,210), Italy (to Bianca De Filippis), and by Peretti foundation.

## CONFLICT OF INTEREST

The authors confirm that there are no conflicts of interest.

## Supporting information


FigureS1

FigureS2

FigureS3

FigureS4

## Data Availability

The data supporting the findings of this study are available from the corresponding authors upon reasonable request
