# Supplementary material for: Effects of the Rho GTPase‐activating toxin CNF1 on fibroblasts derived from Rett syndrome patients: A pilot study
Source: J Cell Mol Med. 2023 Apr 20;27(10):1315–26. doi: 10.1111/jcmm.17624 (PMC10183712; doi:10.1111/jcmm.17624)
Supplement: Supplementary file 1 — FigureS1 [file JCMM-27-1315-s001.docx]

Supplementary Figure 1. Clinical features (table A) and birth data (table B)

| **Table A - Clinical features** | | | | | | | | | |
| --- | --- | --- | --- | --- | --- | --- | --- | --- | --- |
| **ID** | **Mutation** | **Age of onset / regression** | **Seizures** | **Word acquisition** | **Language / communication** | **Ambulation** | **Hand use** | **Instrumental exams** | **Other features** |
| RTT1  2205 | MECP2 late truncating mutation  MECP2 A443X | 18 – 20 months | Yes,  at 30 months. | Lallation before 18 months. | At the age of 7 yo, she has never pronounced words or phrases.  She had not been able to speak in sentences nor in single words.  Sporadic lallation. | Deambulation at 18 months.  Gait with a wide base of support.  Swaying movements to the side and forward-backward. | Hand stereotypies.  At the age of 7 yo, she had not grabbed objects. | EEG: normal  Brain CAT:  right temporo-occipital leukomalacia | Hyperventilation, apnea, sialorrhea, bruxism, motor stereotypes of the hands, prognathism, cold hands and feet.  Physical examination at 7 yo: weight 18 kg (5-10° percentile), height 122 cm (50-75° percentile), head circumference 49 cm (3° percentile) |
| RTT2  2282 | c.1163-1201del38  deletion of exons 3 and 4 | 18 months | NA | Lallation at 10 months. | Vocalizations. | At 13-14 months, she had not the ability to walk or sit and she begins psychomotricity. At 15 months she was able to sit, say 3 words (mom, dad and hello), purposefully use her hands. Movement at the midline at 18 – 20 months. | Stereotypical hand wringing. | Brain CAT: normal | Difficulty in swallowing.  Improved sleep disturbances following the intake of risperidone for 12 months.  Since 2003, severe self-injurious behavior has occurred (slapping or punching herself).  Breathing disturbances (probably apnea). |
| RTT3  1425 | c.1163-1201del38  preserved speech communication variant | At about 14 months, regression in fine motor skills (turning the pages of a book or grabbing  little objects). | No | Verbal communication at 18 months. | She is able to speak in simple  sentences. | Deambulation at 18 months, no delays in sitting. | Scarce stereotypical hand wringing movement.  ­­­­­ | NA | She is able to eat alone.  Bruxism, cold extremities (especially the feet), apnea during sleep, not reported gastroesophageal reflux nor intestinal function imbalance, toilet trained (except for sporadic nocturnal enuresis), regular sleep routine.  Weight 49 kg, height 129 cm, head circumference 54 cm |
| RTT4  729 | c.1163-1201del38  MECP2 late truncating mutation  MECP2 R294X | At 24 months,  stereotypies following mononucleosis (fever and yellow vomit). | No | Lallation at 10 months. | She says “mama”.  Vocalizations. | Deambulation at 15 months. | NA | EEG: minor slowing, mainly on the right | NA |

| **Table B - Birth data** | | | | | |
| --- | --- | --- | --- | --- | --- |
| **ID** | **Date of birth** | **Weight at birth (g)** | **Lenght at birth (cm)** | **Head circumference (cm)** | **Apgar score (1’ and 5’)** |
| RTT1 - 2205 | March 11th, 2003 | NA | NA | NA | NA |
| RTT2 - 2282 | April 22th, 2008 | 2970 | 48,5 | 34 | 9 and 10 |
| RTT3 - 1425 | June 26th, 1998 | 3330 | 51 | 34 | NA |
| RTT4 - 729 | June 5th, 1992 | 3500 | 50 | 34 | 8 and 8 |
