# Supplementary figures and images for: Effects of the Rho GTPase‐activating toxin CNF1 on fibroblasts derived from Rett syndrome patients: A pilot study
Source: J Cell Mol Med. 2023 Apr 20;27(10):1315–26. doi: 10.1111/jcmm.17624 (PMC10183712; doi:10.1111/jcmm.17624)

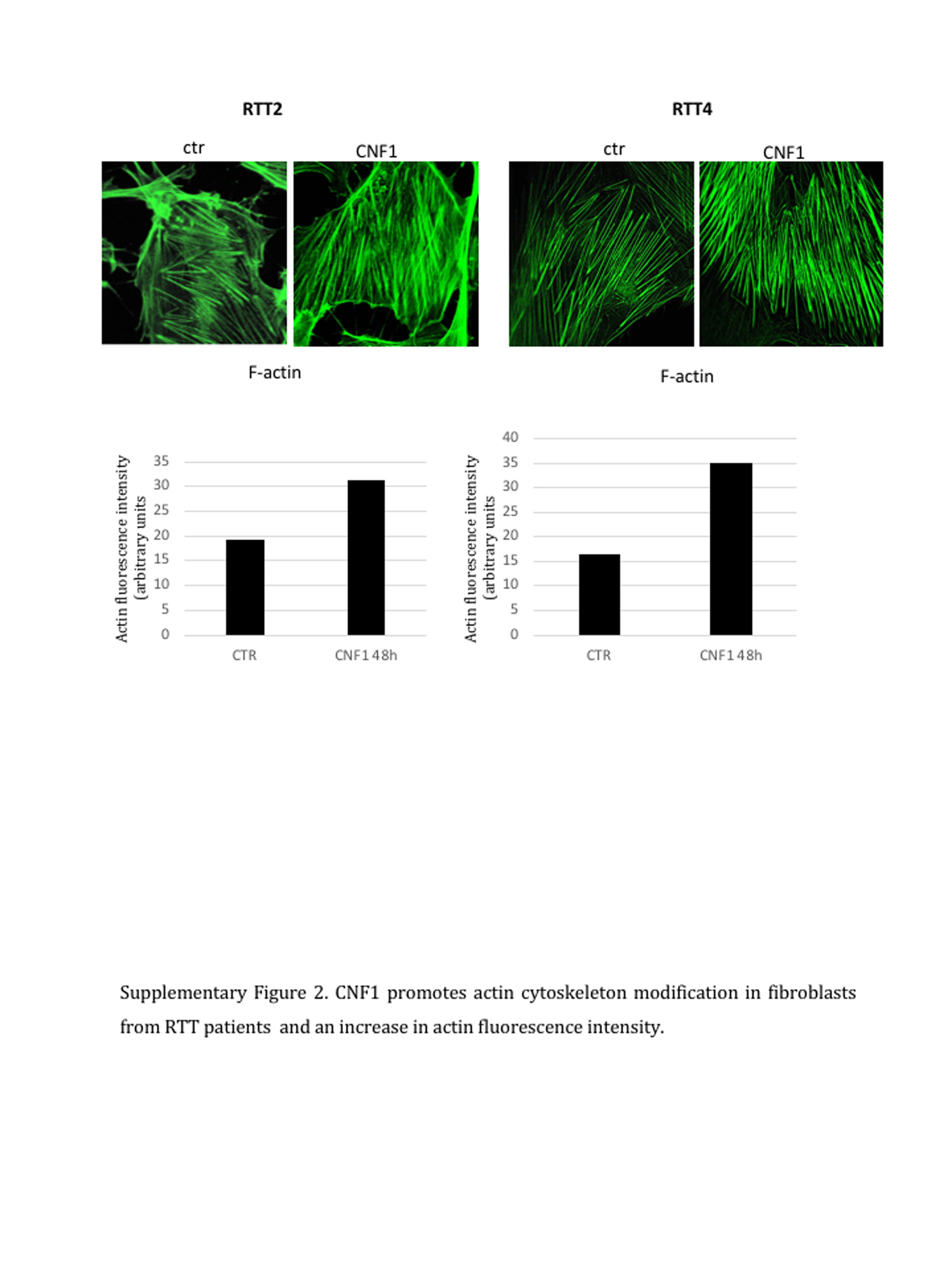

Supplement: Supplementary file 2 — FigureS2 [file JCMM-27-1315-s003.tif]

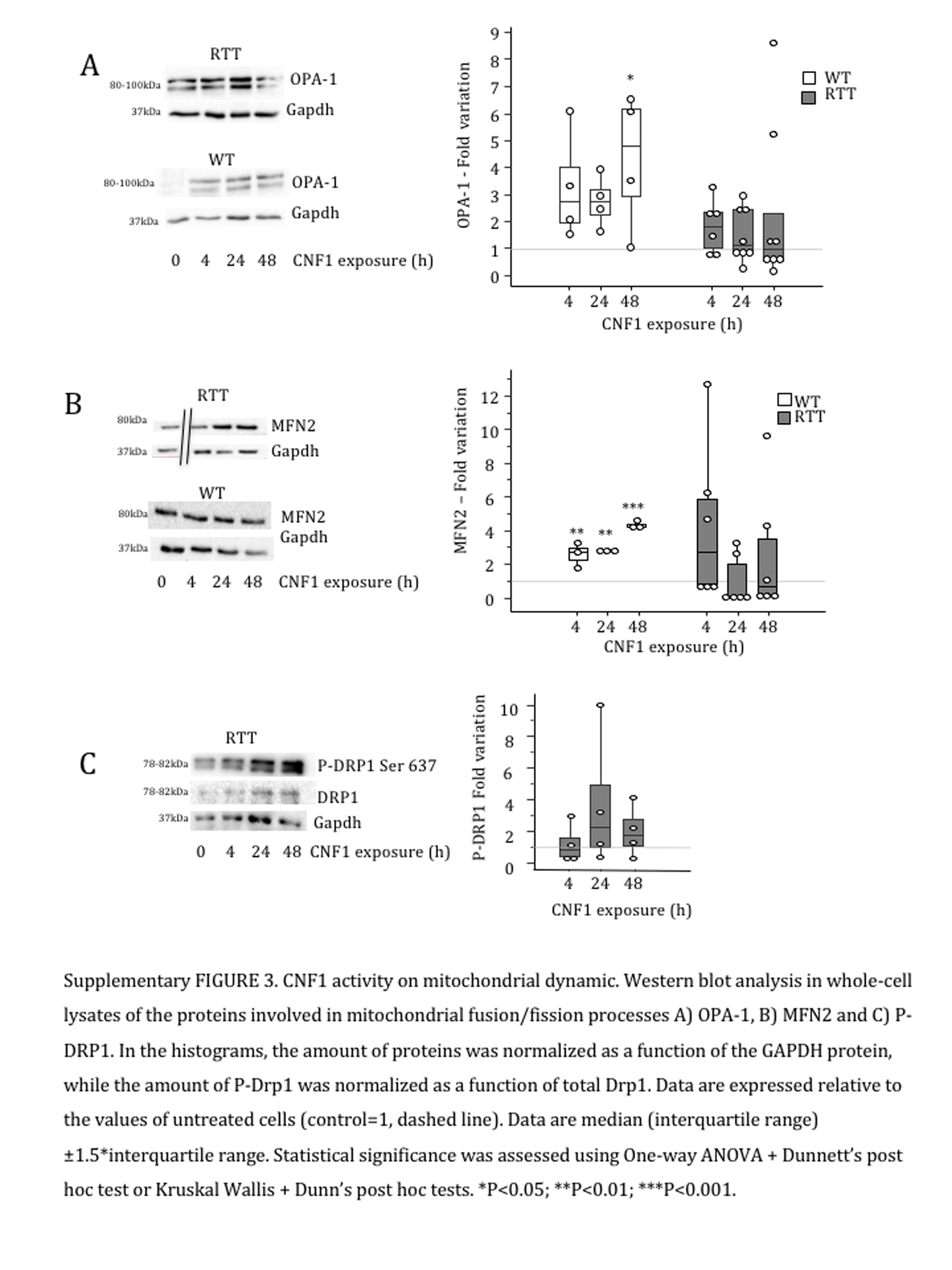

Supplement: Supplementary file 3 — FigureS3 [file JCMM-27-1315-s002.tif]

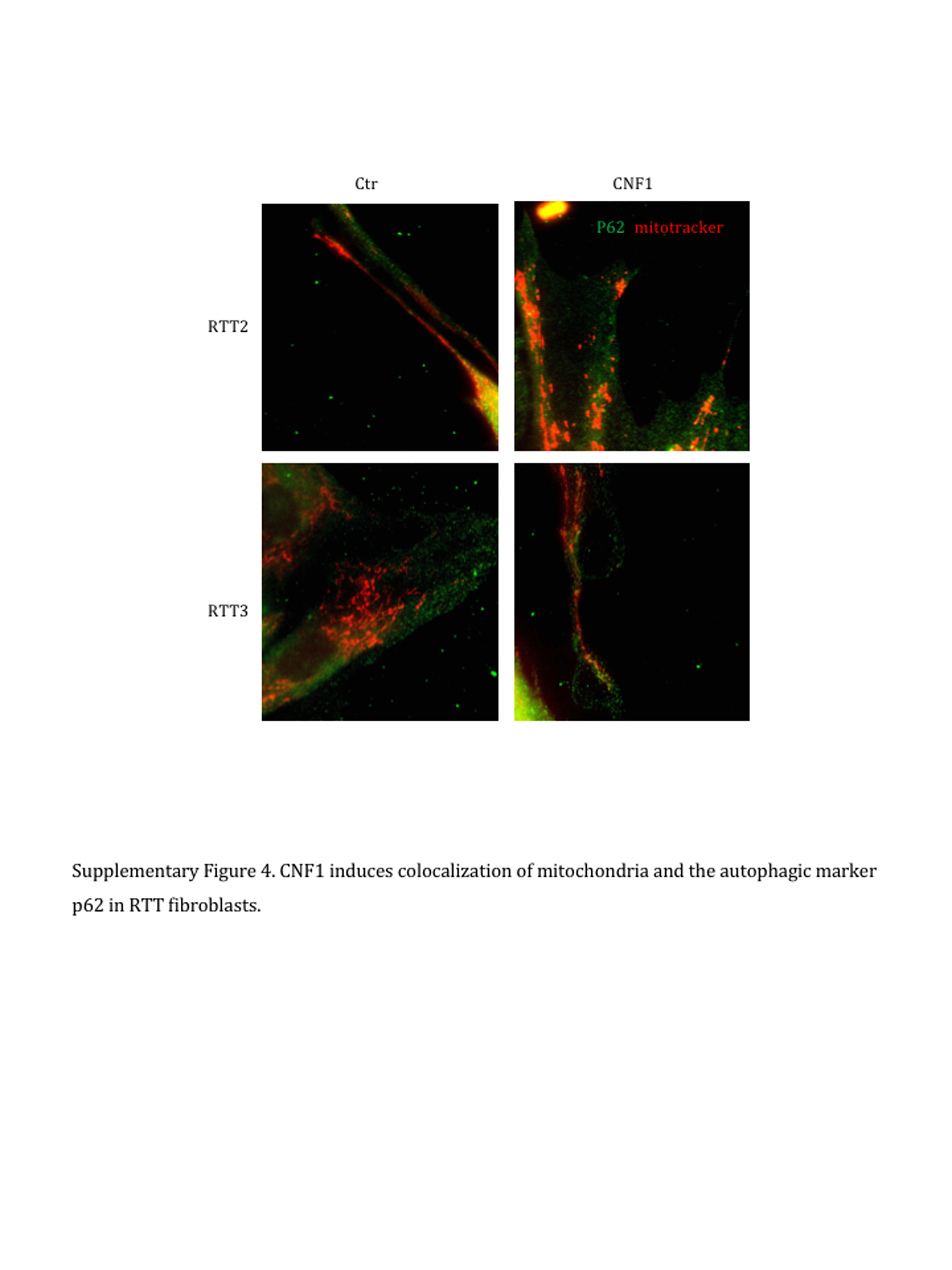

Supplement: Supplementary file 4 — FigureS4 [file JCMM-27-1315-s004.tif]
